# Preliminary Term in Buffalo Medical College

**Published:** 1874-08

**Authors:** 


### Preliminary Term in Buffalo Medical College.

It will be observed by notice of our Advertisement of this College, that a very important addition has been made to the instruction of the *preliminary term*, as it is called. It has been customary to devote all the time to Dissections, except the Clinical Lectures given in the Hospital, but Students tire of the labors of a dissecting room and soon hurry over the work wanting to ge it off their hands as soon as possible. It is believed that by listening two hours a day to lectures, nothing will be lost to the Dissecting term, and that great gain will be obtained to the departments in which didactic lectures are given. It is practically lengthening the College term, without increasing its expenses, and giving increased facilities for complete Medical Education in an Institution which now stands at the very head of American Medical Colleges.

Those familliar with the course of instruction in the Buffalo Medical College, and with most other similar institutions of the world, do not hesitate to say, that in no country, is Medicine and Surgery more thoroughly or more practically taught, than in the University of Buffalo. This is duly appreciated by the profession as shown by the constantly increasing numbers of students who annually graduate at this College, and we have no doubt this will continue as long as the present standard of excellence is maintained.

